# A SEMA3 Signaling Pathway-Based Multi-Biomarker for Prediction of Glioma Patient Survival

**DOI:** 10.3390/ijms21197396

**Published:** 2020-10-07

**Authors:** Indre Valiulyte, Giedrius Steponaitis, Deimante Kardonaite, Arimantas Tamasauskas, Arunas Kazlauskas

**Affiliations:** Neuroscience Institute, Medical Academy, Lithuanian University of Health Sciences, LT-50161 Kaunas, Lithuania; giedrius.steponaitis@lsmuni.lt (G.S.); deimante.kardonaite@stud.lsmu.lt (D.K.); arimantas.tamasauskas@lsmuni.lt (A.T.); arunas.kazlauskas@lsmuni.lt (A.K.)

**Keywords:** glioma, glioblastoma, semaphorin, SEMA3, neuropilin, plexin, VEGF, gene expression, patient survival, biomarker

## Abstract

Glioma is a lethal central nervous system tumor with poor patient survival prognosis. Because of the molecular heterogeneity, it is a challenge to precisely determine the type of the tumor and to choose the most effective treatment. Therefore, novel biomarkers are essential to improve the diagnosis and prognosis of glioma tumors. Class 3 semaphorin proteins (SEMA3) play an important role in tumor biology. SEMA3 transduce their signals by using neuropilin and plexin receptors, which functionally interact with the vascular endothelial growth factor-mediated signaling pathways. Therefore, the aim of this study was to explore the potential of SEMA3 signaling molecules for prognosis of glioma patient survival. The quantitative real-time PCR method was used to evaluate mRNA expression of *SEMA3(A-G)*, neuropilins (*NRP1* and *NRP2*), plexins (*PLXNA2* and *PLXND1*), cadherins (*CDH1* and *CDH2*), integrins (*ITGB1, ITGB3, ITGA5,* and *ITGAV*), *VEGFA* and *KDR* genes in 59 II-IV grade glioma tissues. Seven genes significantly associated with patient overall survival were used for multi-biomarker construction, which showed 64%, 75%, and 68% of accuracy of predicting the survival of 1-, 2-, and 3-year glioma patients, respectively. The results suggest that the seven-gene signature could serve as a novel multi-biomarker for more accurate prognosis of a glioma patient’s outcome.

## 1. Introduction

Gliomas are the most common and lethal central nervous system (CNS) tumors with unfavorable patient survival prognosis. The incidence rate of glioma patients is about 7.3 per 100,000 persons a year, which is higher in men than in women (8.7 cases and 5.9 cases, respectively). The peak of incidence is reached at the age of ≥65 years [1]. Among glioma tumors, diffuse glioma is the most common type, according to WHO classification 2016, graded as WHO grade II (diffuse), WHO grade III (anaplastic) and WHO grade IV (glioblastoma) [2,3]. Glioblastoma (GBM) is a very aggressive and malignant tumor, which exhibits differentiated and undifferentiated cells with multiple genetic alterations and variations [4,5]. Because of the molecular heterogeneity, it is difficult to determine the type and behavior of the tumor since tumor cells spread quickly and infiltrate into the surrounding tissues. It is noteworthy that some of the tumor cells may be resistant to therapy, and drugs used for treatments can lead to side effects [5]. Despite the complex treatment (surgery, chemotherapy, and radiation therapy), the survival time of patients is generally short (approximately 14 months after initial diagnosis) [6].

Genetic profiling of gliomas using genetic, epigenetic, and transcriptome analysis techniques allows for a more objective and accurate assessment of histopathologic diagnosis, the determination of tumor type and supposed response to treatment [7,8,9]. Therefore, several important glioma biomarkers were included into the updated WHO classification system as potential therapeutic targets: *MGMT* (O6-methylguanine DNA methyltransferase) methylation, *IDH1/2* (isocitrate dehydrogenase 1/2) mutations, *EGFR* (epidermal growth factor receptor) amplification, *TERT* (telomerase reverse transcriptase) promoter mutations, and *PTEN* (phosphatase and tensin homolog) deletions [2,9,10]. These biomarkers improved the classification of the tumor, allowed to predict the outcome of patients more accurately and to select the most effective treatment therapy. For example, in adult patients, lower grade (grade II and III) diffuse astrocytic tumors are typically *IDH*-mutant, and many glioblastomas with an *IDH* mutation are defined as secondary GBMs. On the contrary, *IDH*-wild type glioblastomas are classified as primary GBM tumors [2]. Patients with the *IDH* mutation have a better prognosis than those without *IDH* mutation [11,12] and drug temozolomide, used for glioma treatment, significantly prolonged survival of patients with hypermethylation of the *MGMT* gene promoter [13]. However, finding a way to predict or to improve the survival of patients remains an important task.

Semaphorins (SEMA) are a large family of proteins, widely expressed in a variety of tissues, mostly in CNS during their development. They play an important role in organ development, tissue repair, immune responses, as well as tumorigenesis processes [14,15]. Based on the protein structure and phylogenetic analysis, SEMA are divided into eight subclasses. Class 1 and 2 proteins are found in invertebrates, class 3, 4, and 7—in vertebrates, class 5 in viruses, and only class 5 SEMA are found in both vertebrates and invertebrates. Class 2, 3, and 5 members are secreted SEMA proteins while class 1, 4, 5 and 6 members are transmembrane proteins, and class 7 are glycosylphosphatidylinositol (GPI)-linked proteins [15,16]. Recently, there have been a growing number of scientific studies demonstrating that secreted class 3 semaphorin proteins (SEMA3) are associated with tumor angiogenesis process, cancer cell proliferation, invasiveness, and metastatic spreading [14,17], making it a promising object in the field of cancer research.

Families of secreted SEMA3 proteins are consisted of seven members (A–G) that form a complex with different neuropilin (NRP1, NRP2) and plexin (PLXNA, PLXND) receptors. SEMA3A interacts with the NRP1 and PLXNA, SEMA3F and SEMA3G proteins interact with NRP2 and PLXNA receptors, whereas SEMA3B, SEMA3C and SEMA3D proteins interact with both neuropilin isoforms (NRP1, NRP2) and the PLXNA receptor. SEMA3E does not interact with the neuropilin, but directly binds to the PLXND1 receptor [14,18]. In addition, SEMA3 transduce their signals by using neuropilin and plexin receptors in complex with integrins [19,20], cadherins [21,22], and vascular endothelial growth factor receptors (VEGFR) [23,24], showing the ability of SEMA3 proteins to activate and regulate a variety of different signaling pathways [15,19]. The formation of these diverse complexes is important for SEMA3 signaling, as they regulate the growth of axons and dendrites in the CNS, angiogenesis, cell migration, invasion, proliferation, and epithelial to mesenchymal transition processes [19,21]. In cancer biology, depending on activity of different SEMA3-receptor complexes, SEMA3 proteins can mediate either tumor-promoting or tumor-suppressing functions [20].

In this study, we aimed to examine expression profiles of genes encoding key members of the SEMA3 signaling pathway in astrocytoma samples of different malignancy grades and, based on the obtained data, to create a multi-biomarker for better patient survival prognosis.

## 2. Results

### 2.1. Associations of Expression Changes of Key SEMA3 Signaling Genes in Astrocytomas with Patient Clinical Data

**Figure 1:**
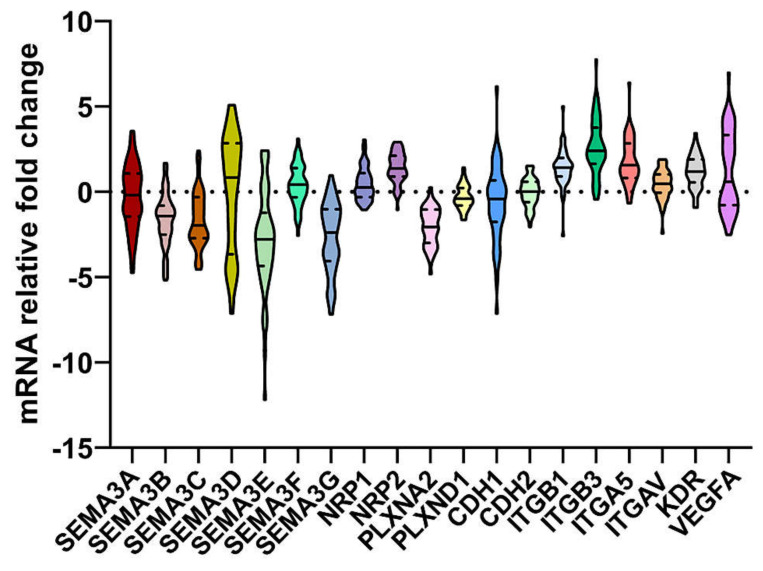
The distribution of mRNA levels of class 3 semaphorin (SEMA3) signaling genes in 59 astrocytoma samples. The solid line inside the violin represents the median, the dotted lines inside the violin represent quartiles. Relative fold change of mRNA is presented as 2^−ΔΔCt^ (The gene expression in normal brain tissue was normalized to 0 point).

Initially, the expression of *SEMA3(A-G), NRP1, NRP2, PLXNA2, PLXND1, CDH1, CDH2, ITGB1, ITGB3, ITGA5, ITGAV, KDR,* and *VEGFA* genes at mRNA level were compared between astrocytoma and healthy human brain (control) samples. As shown in Figure 1, the mRNA levels of all analyzed genes were altered in tumor samples, compared to control (zero point), indicating their importance in gliomagenesis process. The highest fold change was observed in expression of *SEMA3E* and *ITGB3* (the medians of mRNA levels of *SEMA3E* and *ITGB3*: -2.78 and 2.41, respectively). In contrast, the expression of *PLXND1* and *CDH2* genes was slightly dispersed around the zero point (the median of mRNA levels of *PLXND1* and *CDH2*: −0.4 and 0.01, respectively).

The Mann-Whitney U-Test was used to analyze whether expression of analyzed genes were associated with patient gender, age at the time of surgery, malignancy grade of astrocytoma, *IDH* mutation and *MGMT* gene methylation status (Table 1).

**Figure 2:**
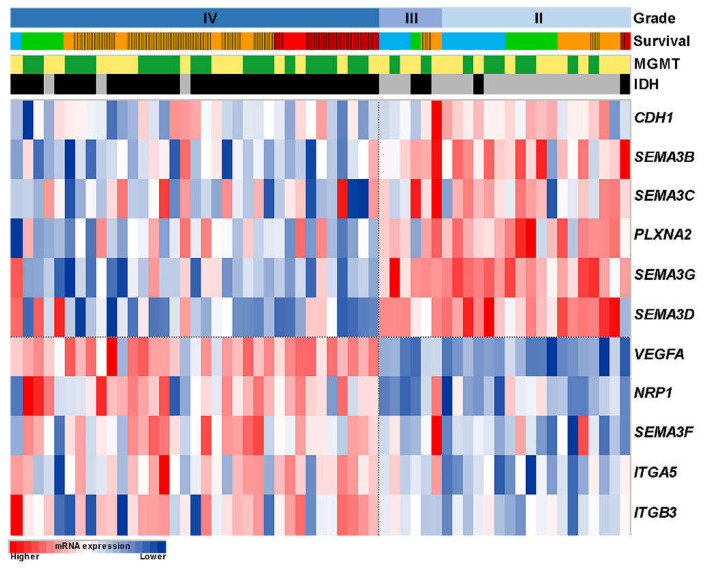
The heatmap of mRNA levels of SEMA3 signaling members in astrocytomas. Expression levels of 11 (out of 19) genes were significantly associated with the tumor grade (Kruskal-Wallis Test, *p* < 0.05). Survival (patient survival time after surgical resection of the tumor): red color—patients survived less than 1-year, orange—patients survived 1-year, green—survived 2-years, and blue—survived 3-years, strait lines indicate patients who are dead, others are censored; *MGMT*: yellow color—methylated, green—unmethylated; *IDH*: grey—mutation, black—wild-type.

Our results revealed that men compared to women had statistically higher expression levels of *SEMA3B, SEMA3D, SEMA3G,* and *PLXNA2* genes (*p* < 0.05). Based on the patient age average (50 year), patients were divided into two groups: patients who were younger than 50 year (≤ 50) and patients who were older than 50 years (> 50). The statistical analysis showed increased mRNA levels of *SEMA3A, SEMA3F, NRP1, ITGB3, ITGA5,* and *VEGFA* in >50-year patient group (*p* < 0.05). In contrast, expressions of *SEMA3B, SEMA3D, SEMA3G, NRP2, PLXNA2, CDH1,* and *ITGAV* genes were increased in ≤50-year patient group (*p* < 0.05). In relation to the malignancy grade of the tumor, mRNA levels of *SEMA3B, SEMA3C, SEMA3D, SEMA3G, PLXNA2*, and *CDH1* decreased while mRNA levels of *SEMA3F, NRP1, ITGB3, ITGA5*, and *VEGFA* increased with the increase of the tumor malignancy (*p* < 0.05) (Table 1). A heatmap was used to vividly show the gene expression profiles of 11 genes, significantly associated with malignancy grade of astrocytoma (Figure 2). Among the tumor specimens with *IDH* mutation, mRNA levels of *SEMA3B, SEMA3C, SEMA3D, SEMA3G, NRP2, PLXNA2,* and *CDH1* were significantly higher (*p* < 0.05), while *SEMA3F, NRP1, ITGB3, ITGA5,* and *VEGFA* genes were under-expressed (*p* < 0.05). Methylated *MGMT* gene was associated with higher *SEMA3C* (*p* < 0.01) levels and lower *SEMA3F, VEGFA,* and *KDR* (VEGF receptor) expression levels (*p* < 0.05) (Table 1).

**Figure 3:**
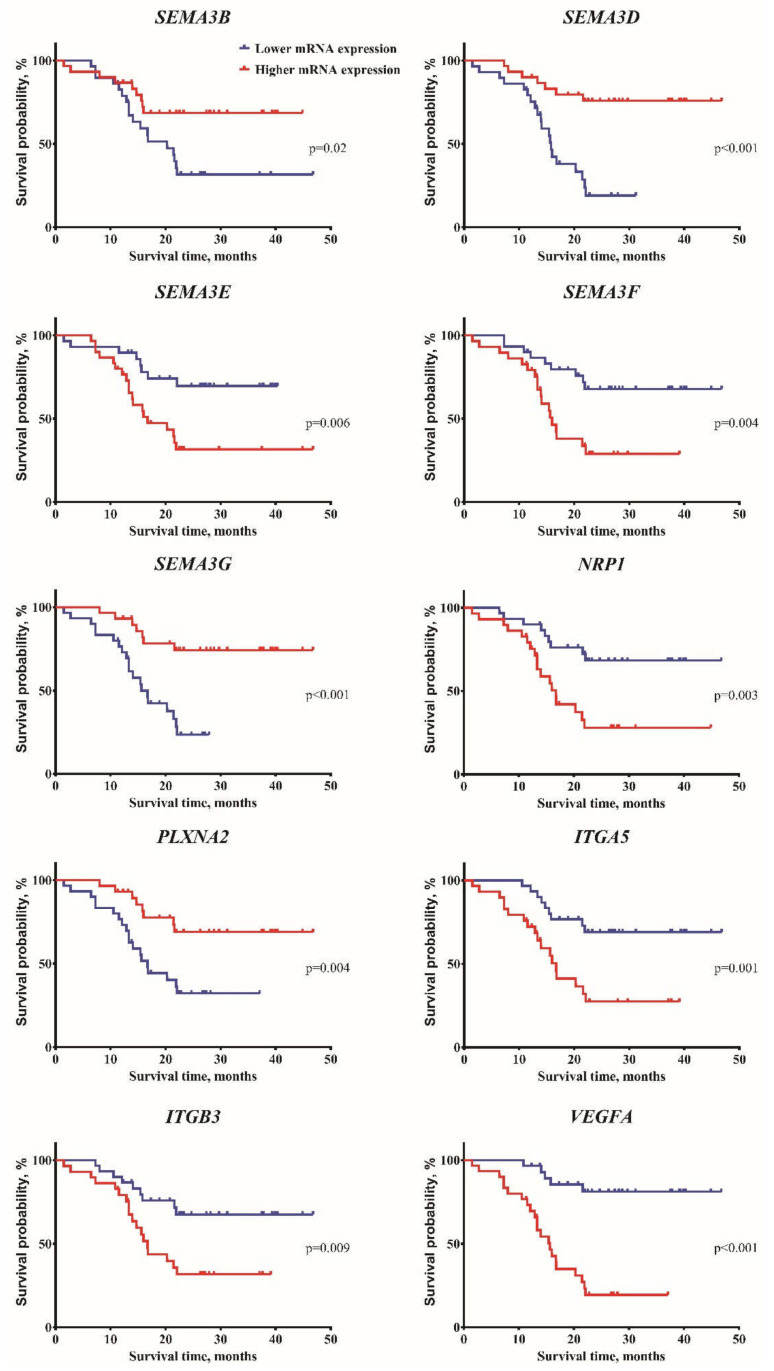
Kaplan-Meier survival curves of SEMA3 pathway genes. Astrocytoma patients (n = 59) divided into two mRNA expression groups (lower and higher) according to the median of the gene expression. Log-rank test, *p* < 0.05.

To determine if expression changes of SEMA3 signaling pathway genes were associated with patient survival, all analyzed genes were divided into lower (≤median value) and higher (>median value) gene expression groups, according to the median values of previously determined (Figure 1) mRNA levels. Kaplan-Meier analysis with log-rank test revealed that patients who had higher mRNA levels *of SEMA3B, SEMA3D, SEMA3G,* and *PLXNA2* had a statistically significant higher rate of survival time (χ^2^: 5.42, 15.71, 13.44, and 8.18, respectively, *p* < 0.05) (Table 1 and Figure 3). Half of patients with lower expression of these genes died within 20.27 months (95% CI: 12.67–27.87), 15.8 months (95% CI: 13.56–18.04), 15.64 months (95% CI: 12.31–18.97), and 16.75 months (95% CI: 14.51–19), respectively. The significantly higher rate of patient survival was associated with lower mRNA levels of *SEMA3E, SEMA3F, NRP1, ITGA5, ITGB3,* and *VEGFA* genes (χ^2^: 7.56, 8.45, 8.99, 10.23, 6.84, and 22.03, respectively, *p* < 0.01) (Table 1 and Figure 3). Fifty percent of patients in the higher expression group of these genes died within 16.75 months (95% CI: 9.04–24.46), 16 months (95% CI: 12.89–19.12), 16.75 months (95% CI: 13.46–20.04), 16.75 months (95% CI: 14.9–18.6), 16.75 months (95% CI: 13.67–19.83), and 15.64 months (95% CI: 12.41–18.87), respectively.

By summarizing results, we noticed that *SEMA3B, SEMA3D, SEMA3F, SEMA3G, NRP1, PLXNA2, ITGB3, ITGA5,* and *VEGFA* were significantly associated with 4 clinicopathological characteristics (age, tumor grade, *IDH* mutation and survival time), which were closely related to unfavorable patient outcomes (genes in bold in Table 1). Lower mRNA expression of *SEMA3B, SEMA3D, SEMA3G,* and *PLXNA2* or higher mRNA expression of *SEMA3F, NRP1, ITGB3, ITGA5,* and *VEGFA* genes were detected in the older patient group and associated with the higher tumor grade, wild-type status of *IDH*, and lower rate of survival time (*p* < 0.05).

Finally, correlation analysis between mRNA levels of all 19 examined genes was performed (Supplementary Table S1). In line with the SEMA3 protein binding preference to particular neuropilin and plexin receptors determined in numerous studies [14,18], *SEMA3B* expression positively corelated with expression of its receptors *NRP2* (r = 0.35, *p* = 0.006) and *PLXNA2* (r = 0.54, *p* < 0.001), *SEMA3C* positively correlated with *NRP2* (r = 0.31. *p* = 0.018), *SEMA3D* positively correlated with *PLXNA2* (r = 0.33, *p* = 0.011) and negatively correlated with *NRP1* (r = −0.46, *p* < 0.001), and *SEMA3G* had the strongest positive correlation with the receptor *PLXNA2* (r = 0.61, *p* < 0.001). These observations justify that SEMA3-receptor complexes are necessary for signaling pathways, involved in cancer-associated processes [14,16]. Interestingly, the strongest positive correlations were observed between *SEMA3B* and *SEMA3G* (r = 0.63, *p* < 0.001), *PLXND1* and *ITGA5* (r = 0.61, *p* < 0.001), *ITGB3* and *ITGA5* (r = 0.66, *p* < 0.001). Contrary, *SEMA3D* inversely correlated with *ITGA5* and *VEGFA* (r = −0.66 and −0.66, *p* < 0.001, respectively), while *SEMA3G* with *VEGFA* (r = −0.65, *p* < 0.001). The cause of mentioned interactions is not clear; therefore, more studies are needed to explain these correlations. It is noteworthy that no correlation (neither positive or negative) between mRNA levels of *VEGFA* and its receptor *KDR* was determined.

### 2.2. Construction of the Multi-Biomarker for Better Patient Survival Prediction

To evaluate the relationship between patient clinicopathological factors (gender, age until operation, glioma malignancy grade, methylation of *MGMT*, and mutation status of *IDH*), and patient overall survival (OS), as well as to determine survival-related genes, an univariate Cox proportional hazard regression analysis was performed on expression data of 19 SEMA3 signaling genes. As shown in Table 2, patient age, tumor grade, status of *IDH* mutation and expression of seven genes (*SEMA3A, SEMA3D, SEMA3F, SEMA3G, ITGB3, ITGA5,* and *VEGFA*) significantly correlated with patient OS (*p* < 0.05). Younger patient group and lower tumor malignancy grade was associated with longer OS (*p* < 0.01). Better OS prognosis was also related to mutation of *IDH*, lower expression levels of *SEMA3A, SEMA3F, ITGB3, ITGA5,* and *VEGFA* or higher expression of *SEMA3D* and *SEMA3G* genes (*p* < 0.05).

Based on the results of univariate Cox regression analysis, seven genes, significantly associated with patient OS, were selected to construct the risk signature. The risk-score formula was constructed as follows (described in “Statistical analysis” section): risk score = (0.237 × *SEMA3A* expression) + ((−0.229) × *SEMA3D* expression) + (0.5 × *SEMA3F* expression) + ((−0.228) × *SEMA3G* expression) + (0.243 × *ITGB3* expression) + (0.412 × *ITGA5* expression) + (0.303 × *VEGFA* expression). Among them, high levels of *SEMA3A, SEMA3F, ITGB3, ITGA5,* and *VEGFA* genes were defined as risky factors (HR > 1), while high levels of *SEMA3D* and *SEMA3G* genes—were defined as protective factors (HR < 1).

**Figure 4:**
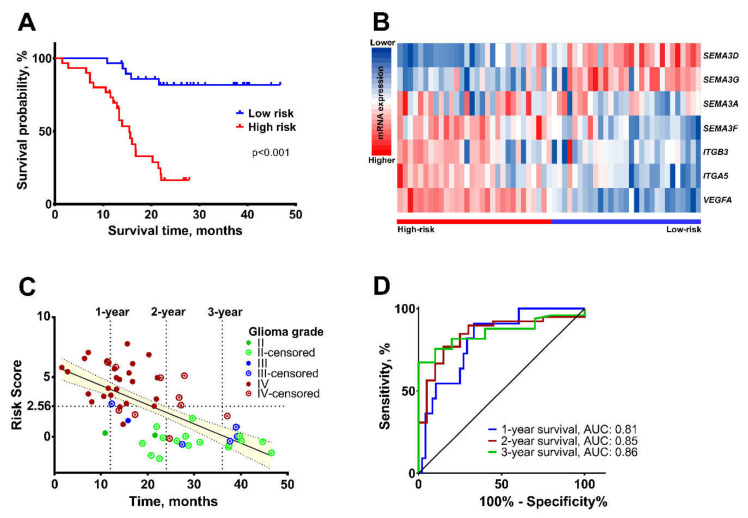
Characteristics of a multi-biomarker in training cohort. (**A**) Kaplan-Meier analysis in high-risk and low-risk patient groups, arranged by seven-gene risk score median; log-rank test, *p* < 0.05. (**B**) The heatmap of mRNA expression distribution of seven genes in high-risk and low-risk patient groups. (**C**) Distribution of patient status with the diagnosis of different malignancy grade glioma tumor, according to survival time and seven-gene risk score. The median risk score value 2.56, r = −0.66, *p* < 0.001. The yellow-shaded area represents 95% confidence interval. (**D**) ROC curves of 1-, 2-, and 3-year patient survival, according to the seven-gene signature.

According to median value of the risk score (2.555), all 59 patients were divided into low-risk (≤ 2.555, *n* = 29) and high-risk (> 2.555, *n* = 30) groups. The Kaplan-Meier analysis with log-rank test was used to compare overall survival between these two patient groups. As shown in Figure 4A, patients in high-risk group had poor prognosis compared to low-risk group (χ^2^ = 24.7, *p* < 0.001). Among these two groups, the higher expression levels of *SEMA3G* and *SEMA3D* genes were noticed in the low-risk group, while *SEMA3A, SEMA3F, ITGB3, ITGA5,* and *VEGFA*—were noticed in the high-risk group (Figure 4B). The dot-plot graph was used to represent the distribution of patient status (alive or dead) with the diagnosis of different malignancy astrocytoma in relationship with patient survival time and risk score (Figure 4C). The correlation analysis showed that lower risk-score was significantly associated with better patient prognosis—longer survival time and low tumor malignancy (r = −0.66, *p* < 0.001). In the high-risk group 13.56% (8/59) of patients died within 1-year and 37.29% (22/59)—within 2-year period, while in the low-risk group 8,47% 5/59 of patients died within 2-years. Furthermore, Mann-Whitney and Kruskal-Wallis tests revealed significant multi-biomarker risk-score associations with astrocytoma pathological grade (*p* < 0.001), patient disease appearance age (≤ 50 and >50 years, *p* < 0.001), *IDH* status (wild-type and mutation, *p* < 0.001), and *MGMT* status (methylated and unmethylated, *p* = 0.027), but not gender (*p* = 0.067) (Table 1). The diagnostic values of the multi-biomarker of selected seven genes for 1-, 2-, and 3-year survival prediction were confirmed with the receiver operating characteristic (ROC) curve analysis (Figure 4D). The calculated areas under the ROC curves (AUC) for 1-, 2-, and 3-year survival were 0.81 (95% CI: 0.685−0.937), 0.85 (95% CI: 0.74−0.95), and 0.86 (95% CI: 0.76−0.952), respectively (*p* < 0.001). The accuracy of 1-, 2-, and 3-year survival prediction were: 64%, 75%, and 68%, respectively.

In addition, to analyze patient survival with respect to several factors simultaneously, the clinical characteristics and genes from univariate Cox regression with significance *p* < 0.05 were sorted out and multivariate Cox regression analysis performed. The analysis revealed that the expression of 3 genes (*Sema3A, Sema3D,* and *ITHB3*) and *IDH* status are independent indicators increasing the risk of patient death. Wild-type *IDH* type increased event risk by 5.54-fold (95% CI: 1.46–21.023; *p* = 0.012), while higher mRNA expression of *SEMA3A* and *ITGB3*, but lower mRNA expression of *SEMA3E* increased event risk by 1.36-, 1.27-, and 0.88-fold, respectively; *p* < 0.05) (Table 2). However, after the multi-biomarker construction using significant variables from multivariate Cox regression test, the patient survival analysis between high- and low-risk patient groups showed no significance (χ^2^ = 0.76, *p* = 0.383) and the accuracy of the model for 1-, 2- and 3-year survival was low (51%, 47% and 47%, respectively, data not-shown). Therefore, the first combination of the multi-biomarker with greater accuracy was used for further analysis.

After demonstrating the survival predicting power of the created signature, the multivariate Cox regression analysis with backwards conditional was performed to clarify the prognostic value in accordance to other clinical features that were significantly associated with patient survival in univariate Cox regression (Table 2). The analysis revealed that the signature remained to be significantly associated with OS when adjusting other clinical factor including age, tumor grade and *IDH* status (HR = 1.27, 95% CI: 1.06–1.54, *p* = 0.012). Importantly, although patient age also showed significant predictive power, the effect was lower compared to the effect of the multi-biomarker (HR = 1.04, 95% CI: 1.00–1.08, *p* = 0.031; Table 2, Multivariate*).

### 2.3. Validation of the Survival Prediction Model

**Figure 5:**
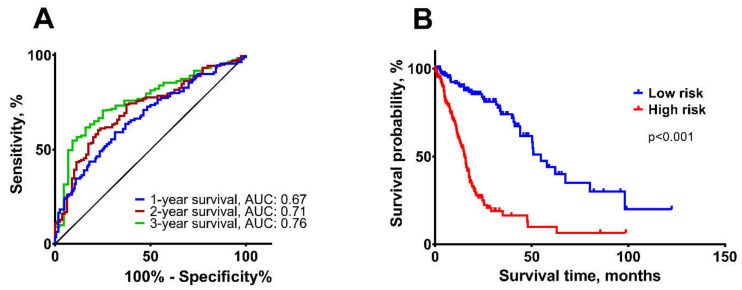
Characteristics of a multi-biomarker in validation cohort. (**A**) ROC curves of 1-, 2-, and 3-year patient survival, according to the seven-gene signature. (**B**) Kaplan-Meier analysis in high-risk and low-risk patient groups, arranged by seven-gene risk score median; log-rank test, *p* < 0.05.

The seven-gene signature was further validated in The Cancer Genome Atlas GBM-LGG dataset via the GlioVis portal [25], including 276 different malignancy grade glioma patients. In this cohort, the risk-score was calculated as described in the multi-biomarker construction section. Mann-Whitney U-test revealed that patients with lower risk-score had methylated *MGMT* (*p* < 0.001), mutant *IDH* status (*p* < 0.001) and were in a younger patient group (≤ 50-year, *p* < 0.001). However, gender showed no significant associations (*p* = 0.714). According to Kruskal Wallis test, higher tumor grade was significantly associated with higher risk-score (*p* < 0.001) (data not-shown). Furthermore, the Cox regression analysis showed that the multi-biomarker was significantly associated with glioma patient OS (HR 4.76, 95% CI: 3.199–7.087, *p* < 0.001). ROC analysis confirmed that the multi-biomarker had a good accuracy of predicting 1-, 2-, and 3-year patient survival (AUC: 1-year 0.67, 95% CI: 0.605–0.737; 2-year 0.71, 95% CI: 0.649–0.778; 3-year 0.76, 95% CI: 0.689–0.830 (Figure 5A). The accuracy of 1-, 2-, and 3-year survival predictions were: 62%, 65%, and 62%, respectively. Finally, according to the Kaplan-Meier analysis, patients in low-risk group had significantly better survival prognosis than in high-risk group (χ^2^ = 69.5, *p* < 0.001). In the low-risk group, 50% of patients died within 54.8 months (95% CI: 46.26-65.34), while in the high-risk group—within 15.4 months (95% CI: 13.304–17.496) (Figure 5B).

## 3. Discussion

Gliomas are rare primary brain tumors (7.3/100,000 cases); nevertheless, very unfavorable functional prognoses and high mortality rates indicate that substantial efforts are necessary to mitigate adverse outcomes of the disease [1]. The 5-year survival rate for the primary CNS cancers is very low (17% for males and 19% for females) [26]. Moreover, glioblastoma, and lower malignancy grade glioma tumor cells have multiple genetic alterations and variations which makes it difficult to precisely determine the type and behavior of the glioma tumor, as well as to choose the most benefiting treatment. Therefore, the newest update of the WHO classification system included several molecular biomarkers for better tumor characterization [2]. In terms of astrocytoma, the updated classification allowed to classify astrocytoma tumors more accurately, as well as, to predict the outcome of patients, and to select treatment strategy, according to the *MGMT* methylation or *IDH* status. However, since astrocytomas exhibit very high inter- and intra-heterogeneity and therefore, the disease course is poorly predictable, the search for novel molecules specifically associated with gliomagenesis still remains an important task for more accurate disease prognosis. Whereas a single marker cannot completely identify the type of glial tumor, as it is in the case of the prostate (PSA gene) [27] or breast cancer (BRCA gene) [28,29], it is crucial to attempt creation of a multi-biomarker with higher predictive value than a single-biomarker, which provides composite biological and therapeutic information about the tumor.

Recently, several studies have shown that the analysis of gene expression profile could detect gene signatures to predict the outcome of patients with glioma tumors. For example, six protein-coding genes (PCG) and five lncRNAs were screened out by a risk score model and a PCG-lncRNA signature was formed that which predicted survival and TMZ-chemoradiation response in GBM patients [30]. Other research groups identified novel biomarkers that have a potential in the outcome prediction of GBM [31,32,33] and which enable to classify patients into high- and low-risk groups based on expression level analysis of the survival relevant genes [34]. Moreover, the novel signature composed of five genes (*DES, RANBP17, CLEC5A, HOXC11,* and *POSTN*) was significantly associated with the 1-, 3-, and 5-year survival of GBM patients, *IDH* status, *MGMT* methylation status, and radio-chemotherapy [35]. However, the clinical applications of these genomic profiles and their molecular mechanisms have not been fully revealed. Considering the poor prognosis of glioma tumors, novel molecular biomarkers are still needed to reveal the mechanisms of gliomagenesis process and to improve patient survival.

The studies of SEMA3 family protein function in glioma tumors are not new in our laboratory. In previous studies, we found that accumulated high SEMA3C protein levels, which did not correlate with mRNA levels, were related to the clinicopathological characteristics of astrocytoma patients [36]. By continuing the previous theme, in our present study, we used different grade astrocytic origin glioma tumor samples to identify SEMA3 signaling pathway genes, significantly associated with gliomagenesis process and patient OS. The prognostic value of SEMA3 signaling pathway members was shown in several studies. Patients with high-VEGF/low-SEMA3 signature had poor prognoses in breast [37] and prostate cancer [38]. Moreover, in the study by Karyian-Tapon and co-authors, higher mRNA expression of *SEMA3B, SEMA3G* and *NRP2* were related to prolonged survival of patients with the diagnosis of glial tumors [39]. In contrast, high *VEGF* expression correlated with higher grade of tumors and poor survival prognosis. However, according to the multivariate Cox analysis, *SEMA3G* and patient age were the only significant prognostic markers associated with a better OS prognosis [39]. In agreement with this study, we also noticed similar tendencies of *SEMA3B, SEMA3G, SEMA3D* and *VEGFA* mRNA level associations with patient survival and tumor malignancy grade. Importantly, our study revealed that expression of other SEMA3 pathway members (*SEMA3F, NRP1, PLXNA2, ITGB3,* and *ITGA5*) were significantly related to unfavorable patient outcomes and may have promising diagnostic value as well (Table 2). The differences between our study and the study by Karyian-Tapon might be because they used different type of tumor specimens (GBM, oligoastrocytoma, and oligodendroglioma) as one study cohort [39], while our study consisted only of astrocytic origin tumors.

To discover a more accurate model for astrocytoma patient survival prognosis, the multi-biomarker was constructed. Since the multivariate Cox regression analysis revealed that the expression of three genes (*Sema3A, Sema3D*, and *ITHB3*) and *IDH* status are independent factors and after the multi-biomarker creation using these variables, the survival analysis between high- and low-risk patient groups showed no significance (χ^2^ = 0.76, *p* = 0.383) and the accuracy of the signature for 1-, 2- and 3-year survival was low (51%, 47%, and 47%, respectively); it was chosen not to follow conventional data analysis steps. The non-standard path for target selection led to the design of the combination of SEMA3 signaling pathways targets for relatively high patient survival accuracy prediction. Therefore, seven genes significantly associated with patient OS of univariate Cox regression analysis were screened out, namely *SEMA3A, SEMA3D, SEMA3F, SEMA3G, ITGB3, ITGA5,* and *VEGFA*. These seven genes were used to construct an independent prognostic multi-biomarker, predicting 1-, 2- and 3-year survival rates (64%, 75% and 68% of accuracy, respectively) of patients with the diagnosis of various malignancies, not for a specific one. To assess the reliability of the model, the novel multi-biomarker was validated in an independent cohort from The Cancer Genome Atlas (TCGA) database via the GlioVis portal (https://gliovis.bioinfo.cnio.es/, accessed August 2019) [25] and similar results were obtained.

By analyzing patient survival in relation to clinical and pathological variables, the results revealed that age, GBM status and *IDH* mutation are good predictors of patient survival [12]. However, they have some limitations. As shown in Figure 4C, in some cases, short survival is observed at a young age (39-year patient survived only 14 months) and GBM patients with poor prognosis survive more than 2- or 3-years. *IDH* status is also useful in predicting the OS of patients diagnosed with GBM, and in the selection of appropriate therapeutic strategies [40,41], however, the majority of grade II and III gliomas have a mutant-type of *IDH* (Figure 2), while survival times vary between these patients (Figure 4C). These exceptions appear due to the heterogeneity of gliomas, which hinders the accurate identification of tumor malignancies. More importantly, the predicting power of the newly created signature was the strongest compared to other clinical characteristics (Table 2, Multivariate*), indicating that the multi-biomarker has a great prognostic value in accordance to other clinical factors and is important for the current clinical management of glioma.

The seven genes of the signature play an important role in molecular pathogenesis of glioma tumors by regulating tumor cell migration, proliferation, invasion, as well as tumor angiogenesis, and immune response [42]. It was demonstrated that expression of SEMA3A, SEMA3D, and SEMA3F proteins significantly inhibited angiogenesis process and the formation of subcutaneous tumors derived from glioblastoma cells U87MG [43]. The overexpression of SEMA3G in U251MG cells inhibited tumor cell migration and invasion in vitro [44], and suppressed angiogenesis process, when SEMA3G overexpressing U87MG cells were implanted in the brain cortex of mice [43]. Integrins ITGA5 and ITGB3 participate in angiogenesis process by regulating endothelial cell migration and survival [45]. It was showed that ITGA5 and ITGB3 are expressed in glioma vessels and tumor cells and the gene expression is significantly associated with the malignancy grade of the tumor and poor patient prognosis [34,46]. Glioblastoma tumors are highly vascularized, therefore the expression of pro-angiogenic factor VEGFA is increased [39]. Importantly, glioma stem cells also produce VEGFA protein which is carried in extracellular vesicles and induce the formation of blood vessels by targeting brain endothelial cells [47]. In summary, all seven genes of the multi-biomarker participate in the regulation of angiogenesis process, which is a key mediator of the tumor microenvironment development, especially of highly vascularized glioma tumors. Therefore, these molecules are of great importance of controlling the process of gliomagenesis.

The findings of this study should be seen in the light of some limitations. One of the limitations is that the increase in the experiment-wise error rate across the reported statistical analyses was not controlled. The correction for multiple testing was not applied since gliomas are known as extremely heterogeneous tumors, consequently, the significant statistical differences could be reduced. Therefore, markers with even a “small” impact are very important to indicate a specific subclass of tumors in an overall combination of signature. Second, the low amount of astrocytoma specimens of training data may result in overfitting that could be resolved by increasing the sample size in biomarker training cohort. Third, due to the relatively small number of tumor samples, it was difficult to investigate the multi-biomarker in the diverse subgroups of gliomas. Therefore, the cohort of tumor samples should be expanded, and follow-up studies performed, to validate the applicability of the biomarker to the prognosis of glioma patients. Overall, we consider this research relatively preliminary and should be critically evaluated.

## 4. Materials and Methods

### 4.1. Data of Patients

The study consisted of 59 patients diagnosed with astrocytic origin glioma tumors of grade II-IV. The malignancy grade was determined according to WHO classification 2016 [2,3]. All patients were operated on at the Department of Neurosurgery, Hospital of Lithuanian University of Health Sciences from April 2015 to April 2018. The written patient consent and permission from Kaunas Regional Biomedical Research Ethics Committee was taken. The clinical data (gender, age at the time of operation, tumor grade) were collected for each patient. The overall survival of the patient was calculated from the date of tumor resection to the date of patient death or database closure (15 March 2019). After surgical resection, tumor samples were frozen in liquid nitrogen. *IDH* mutation (the R132H mutation in the *IDH1* gene) and *MGMT* methylation status of the tumor samples were confirmed by the pathologists. After mRNA expression analysis of SEMA3 pathway members in tumor tissue specimens, genes, significantly associated with patient survival were used to construct a multi-biomarker, which was validated in an independent cohort, consisting of 276 different malignancy grade glioma tumors. The data (expression of SEMA3 pathway genes and housekeeping (*ACTB* and *GAPDH*) genes) were obtained from TCGA GBM-LGG dataset via the GlioVis portal [25].

The clinical characteristics of patients in the training and validation cohorts are shown in Table 3. The patient median age was 50 years (range 24–80 years) in tumor samples cohort and 50 years (range 20–89 years) in the TCGA cohort. The median overall survival time after diagnosis was 20.27 months (range 1.51 to 46.78 months) in training cohort. At the date of the study closure, there were 32/59 censored patients. The median overall survival time in validation cohort was 15.2 months (range 0.1 to 122.5 months) and 150/276 patients were censored.

### 4.2. Total RNA Extraction and cDNA Synthesis

Total RNA was extracted from 59 frozen tumor tissue specimens using mirVana miRNA Isolation Kit (Life Technologies, USA), according to the manufacturer’s instructions. RNA yield and purity were evaluated with spectrophotometer Nanodrop 2000 (Eppendorf, USA). High Capacity cDNA Reverse Transcription Kit (Applied Biosystems, USA) was used for cDNA synthesis. All samples were stored at −80 °C until gene mRNA expression analysis.

### 4.3. Quantitative Real-Time PCR

Quantitative real-time PCR (qRT-PCR) with SYBR green fluorescent dye was performed to analyze mRNA expression of semaphorins *SEMA3(A-G)*, neuropilins (*NRP1* and *NRP2*), plexins (*PLXNA2* and *PLXND1*), cadherins (CDH1 (E-cadherin) and *CDH2* (N-cadherin)), integrins (*ITGB1*, *ITGB3*, *ITGA5* and *ITGAV*), *VEGFA* and VEGF receptor (*KDR*) genes in 59 II-IV malignancy grade gliomas and healthy human brain RNA samples “FirstChoice Human Brain Reference RNA” (Ambion, Life Technologies, USA). Housekeeping genes *ACTB* and *GAPDH* were used as an endogenous control. All primer sequences of analyzed genes, primer melting temperatures and amplicon lengths are shown in Supplementary Table S2. The qRT-PCR was carried out in a total reaction volume of 12 µL: 6 µL of Power SYBR Green PCR Master Mix dye (ThermoFisher Scientific, USA), 15 ng of cDNA sample, and 0.2 µM of each primer and nuclease-free water. Amplification was executed with a Real-Time PCR System “Applied Biosystems 7500 Fast” (Applied Biosystems, USA). The cycling of qRT-PCR was as follows: 10 min at 95 °C for polymerase activation; cycling of 40 cycles: denaturation at 95 °C for 15 s, annealing 58–61 °C (depending on the gene analyzed) for 30 s and elongation at 72 °C. Melting curve was generated for each run. Comparative 2^−∆∆Ct^ method was used to evaluate analyzed gene expression in tumor samples compared to healthy brain tissue.

### 4.4. Statistical Analysis

GraphPad Prism (version 7.0, Graph-Pad Software, Inc., San Diego, CA, USA) and SPSS software (version 25.0, IBM SPSS, Armonk, NY, USA) were used to evaluate the experimental results. The Mann-Whitney U-test was applied to evaluate the statistical significance of gene mRNA expression changes in groups of patient gender, age, *IDH* mutation, or *MGMT* gene methylation status. Differences in gene mRNA expression between malignancy grade of the tumor were analyzed by Kruskal-Wallis Test. The survival time distribution between gene expression groups was assessed with Kaplan-Meier analysis and Log-rank test. Pearson’s correlation coefficient (r) was used to evaluate correlations between gene expression levels. Univariate and multivariate (with backward conditional method) Cox proportional hazards regression analyses were applied to assess the relationships between clinical and molecular variables, and patient’s overall survival. According to the output of univariate Cox regression analysis, the risk score evaluation formula was composed to create a multi-biomarker for patient survival prognosis: Risk score = (coef. β gene 1 × expr. gene 1) + (coef. β gene 2 × expr. gene 2) + (coef. β gene n × expr. gene n) [35], where the regression coefficient (β) and the expression level (expr) of the gene was derived from the univariate Cox regression model. Time-dependent receiver operating characteristic (ROC) curve analysis was applied to assess the prognostic value of the multi-biomarker for 1-, 2- and 3-year patient survival. Areas under the curves (AUC) were calculated along with 95% confidence interval (CI) and higher AUC was associated with a better model of the risk prediction [48]. The accuracy was calculated as follows: Accuracy = (TN + TP)/(TN + TP + FN + FP) = (Number of correct assessments)/Number of all assessments), where TP—true positive, TN—true negative, FN—false negative, and FP—false positive [49]. The level of significance was *p* < 0.05.

## 5. Conclusions

In conclusion, the analysis of mRNA expression of SEMA3 signaling pathway members provided new insights into the pathogenesis and prognosis of glioma tumors. The constructed biomarker of seven genes may have a promising practical value to predict the 1-, 2-, and 3-year patient survival. However, more detailed experiments are needed to confirm the prognostic value of multi-biomarker in glioma patients and to examine how this multi-biomarker is associated with the clinical treatment responses.

## Figures and Tables

**Table 1:** Relationships between expression of SEMA3 genes and patient clinical data.

Genes	Gender			Age (Years)			Tumor Grade				*IDH*			*MGMT*			Survival
	Female, Median	Male, Median	*p* #	≤50, Median	>50, Median	*p* #	II, Median	III, Median	IV, Median	*p* *	Wt, Median	Mut, Median	*p* #	M, Median	U, Median	*p* #	Log-Rank, *p*
*SEMA3A*	−0.12	−0.54	0.867	−0.77	0.79	**0.024**	−0.6	−1.08	0.69	0.073	0.14	−0.54	0.32	−0.04	−0.39	0.952	0.181
***SEMA3B***	−1.81	−0.99	**0.049**	−0.98	−2.06	**0.001**	−0.79	−0.63	−2.36	**<0.001**	−2.03	−0.86	**<0.001**	−1.24	−1.64	0.154	**0.02**
*SEMA3C*	−2.22	−1.37	0.099	−1.4	−2.54	0.089	−0.88	−1.25	−2.6	**0.023**	−2.51	−0.98	**<0.001**	−1.05	−2.55	**0.006**	0.095
***SEMA3D***	−0.89	1.74	**0.031**	2.79	−1.98	**<0.001**	2.89	2.81	−1.94	**<0.001**	−1.98	2.86	**<0.001**	1.26	−0.26	0.237	**<0.001**
*SEMA3E*	−3.36	−2.4	0.072	−3.33	−2.2	0.071	−2.79	−3.43	−2.64	0.815	−2.55	−3.33	0.393	−3.45	−2.45	0.15	**0.006**
***SEMA3F***	0.64	0.08	0.24	−0.11	0.71	**0.006**	−0.24	0.08	0.76	**0.001**	0.71	−0.15	**0.004**	−0.11	0.69	**0.017**	**0.004**
***SEMA3G***	−3.58	−1.74	**0.006**	−1.28	−3.57	**<0.001**	−0.96	−0.96	−3.59	**<0.001**	−3.58	−1.19	**<0.001**	−1.95	−3.34	0.371	**<0.001**
***NRP1***	0.52	0.14	0.189	−0.12	0.73	**0.002**	−0.14	−0.44	0.78	**<0.001**	0.65	−1.12	**0.006**	0.24	0.26	0.617	**0.003**
*NRP2*	1.69	1.29	0.436	1.74	0.98	**0.011**	1.79	1.79	1.14	0.086	1.23	1.66	**0.043**	1.66	1.14	0.262	0.300
***PLXNA2***	−2.49	−1.37	**0.003**	−1.37	−2.32	**0.001**	−1.17	−1.47	−2.35	**<0.001**	−2.34	−1.04	**<0.001**	−1.81	−2.09	0.413	**0.004**
*PLXND1*	−0.36	−0.43	0.976	−0.43	−0.33	0.585	−0.35	−0.34	−0.47	0.902	−0.29	−0.47	0.419	−0.48	−0.2	0.214	0.371
*CDH1*	−0.14	−0.63	0.541	−0.03	−1.23	**0.044**	0.39	−0.98	−1.1	**0.029**	−1.14	0.06	**0.017**	−1.08	−0.05	0.628	0.065
*CDH2*	−0.07	0.13	0.384	0.01	0.02	0.606	−0.01	−0.4	0.13	0.27	0.09	−0.04	0.698	−0.06	0.24	0.172	0.605
*ITGB1*	1.56	1.38	0.169	1.42	1.45	0.952	1.25	0.95	1.6	0.109	1.52	1.31	0.22	1.4	1.52	0.773	0.841
***ITGB3***	2.62	2.32	0.963	2.15	3.08	**0.013**	1.99	2.1	3.61	**0.005**	2.95	2.05	**0.017**	2.31	2.43	0.74	**0.009**
***ITGA5***	1.69	1.41	0.625	1.19	2.27	**0.003**	0.811	1.31	2.28	**0.001**	2.27	1.03	**<0.001**	1.26	1.71	0.182	**0.001**
*ITGAV*	0.16	0.64	0.096	0.77	0.19	**0.022**	0.84	0.7	0.24	0.145	0.25	0.68	0.157	0.43	0.53	0.988	0.451
***VEGFA***	2.22	−0.09	0.063	−0.68	2.95	**<0.001**	−1.07	−0.64	2.94	**<0.001**	2.83	−0.78	**<0.001**	−0.06	2.61	**0.008**	**<0.001**
*KDR*	1.12	1.39	0.123	0.92	1.37	0.66	0.86	1.14	1.36	0.769	1.34	0.89	0.975	0.91	1.68	**0.012**	0.574
The signature	3.68	1.36	0.067	0.01	4.34	**<0.001**	−0.39	0.41	4.62	**<0.001**	4.34	−0.33	**<0.001**	0.96	3.38	**0.027**	**<0.001**

Data are presented as the relative fold change of mRNA expression. Significant associations *p* < 0.05 indicated in bold numbers, # Mann-Whitney U Test, * Kruskal-Wallis Test. Wt—wild-type, Mut—mutation, M—methylated, U—unmethylated; genes in bold are significantly associated with 4 clinicopathological characteristics (age, tumor grade, *IDH* mutation and survival).

**Table 2:** Univariate and multivariate Cox regression analysis in training cohort.

Univariate					Multivariate				Multivariate *			
	HR	95% CI		*p*	HR	95% CI		*p*	HR	95% CI		*p*
		Lower	Upper			Lower	Upper			Lower	Upper	
**Sex**												
Male vs. Female	1.075	0.503	2.298	0.852								
**Age** (years)	1.072	1.041	1.105	**<0.001**	-	-	-	-	1.042	1.004	1.082	**0.031**
**Tumor grade**												
II	1											
III	1.723	0.156	19.010	0.657								
IV (GBM)	11.825	2.769	50.495	**0.001**	-	-	-	-	-	-	-	-
***MGMT***												
M vs. U	0.511	0.234	1.117	0.092								
***IDH***												
Mut vs. Wt	0.104	0.031	0.350	**<0.001**	5.540	1.460	21.023	**0.012**	-	-	-	-
**Gene expression**												
*SEMA3A*	1.268	1.003	1.602	**0.047**	1.361	1.114	1.664	**0.003**				
*SEMA3B*	0.795	0.629	1.007	0.057								
*SEMA3C*	0.781	0.591	1.033	0.083								
*SEMA3D*	0.795	0.719	0.880	**<0.001**	0.876	0.771	0.995	**0.041**				
*SEMA3E*	1.096	0.958	1.253	0.184								
*SEMA3F*	1.648	1.166	2.330	**0.005**	-	-	-	-				
*SEMA3G*	0.796	0.676	0.938	**0.006**	-	-	-	-				
*NRP1*	1.347	0.969	1.873	0.076								
*NRP2*	0.739	0.497	1.100	0.136								
*PLXNA2*	0.774	0.565	1.062	0.112								
*PLXND1*	1.427	0.842	2.417	0.186								
*CDH1*	0.925	0.781	1.094	0.363								
*CDH2*	0.741	0.460	1.192	0.217								
*ITGB1*	0.978	0.702	1.363	0.896								
*ITGB3*	1.276	1.047	1.555	**0.016**	1.270	1.001	1.609	**0.049**				
*ITGA5*	1.509	1.196	1.905	**0.001**	-	-	-	-				
*ITGAV*	0.941	0.624	1.421	0.774								
*VEGFA*	1.354	1.168	1.571	**<0.001**	-	-	-	-				
*KDR*	1.088	0.741	1.599	0.667								
**The signature**												
High vs. low risk	8.308	3.109	22.206	**<0.001**					1.274	1.055	1.539	**0.012**

GBM—glioblastoma, Wt—wild-type, Mut—mutation, M—methylated, U—unmethylated, HR—hazard ratio, CI—confidence interval; numbers in bold show statistical significance, *p* < 0.05. * Multivariate Cox regression analysis of patient clinical characteristics (age, tumor grade, *IDH* status) and constructed biomarker.

**Table 3:** Summary of patient clinicopathological characteristics.

Variables	Tumor samples	Data from TCGA
	N = 59 (100%)	N = 276 (100%)
**Sex**		
Female	26 (44.07)	116 (42.03)
Male	33 (55.93)	160 (57.97)
**Age (years)**		
≤50	29 (49.15)	140 (50.72)
>50	30 (50.85)	136 (49.28)
**Tumor grade**		
II	18 (30.51)	53 (19.20)
III	6 (10.17)	111 (40.22)
IV (GBM)	35 (59.32)	112 (40.58)
***MGMT***		
U	29 (49.15)	106 (38.41)
M	30 (50.85)	170 (61.59)
***IDH***		
Wt	36 (61.02)	154 (55.80)
Mut	23 (38.98)	122 (44.20)

Postoperative astrocytic origin glioma tumors were used for training set and mRNA expression data from The Cancer Genome Atlas GBM-LGG dataset was used for validation set. GBM—glioblastoma, Wt—wild-type, Mut—mutation, M—methylated, U—unmethylated.

## Supplementary Materials

Supplementary Materials can be found at https://www.mdpi.com/1422-0067/21/19/7396/s1. Table S1. Correlations between mRNA expression of genes in glioma tumors. Table S2. Primer sequences of SEMA3 signaling pathway members.

## Abbreviations

GBM	Glioblastoma
CNS	Central Nervous System
WHO	World Health Organization
TCGA	The Cancer Genomic Atlas
SEMA3	Class 3 Semaphorin
NRP	Neuropilin
PLXN	Plexin
CDH	Cadherin
ITG	Integrin
VEGF	Vascular Endothelial Growth Factor
MGMT	O6-methylguanine DNA Methyltransferase
IDH	Isocitrate Dehydrogenase
AUC	Area Under the Curve
