# Supplementary material for: A SEMA3 Signaling Pathway-Based Multi-Biomarker for Prediction of Glioma Patient Survival
Source: Int J Mol Sci. 2020 Oct 7;21(19):7396. doi: 10.3390/ijms21197396 (PMC7582960; doi:10.3390/ijms21197396)
Supplement: Supplementary file 1 [file ijms-21-07396-s001.zip › Supplementary Table S2.doc.docx]

**Supplementary Table S2.** Primer sequences of SEMA3 signaling pathway members

| **Gene** | **Forward 5’ to 3’ orientation** | **Reverse 5’ to 3’ orientation** | **Tm, ᵒC** | **Amplicon size, bp** |
| --- | --- | --- | --- | --- |
| *SEMA3A* | ACCCAACTATCAATGGGTGCCTTA | AACACTGGATTGTACATGGCTGGA | 62 | 159 |
| *SEMA3B* | TCGATCAGCTCCAGGATGTGTTTC | CTTGTGTGCAAAGGGTCCCAAG | 60 | 164 |
| *SEMA3C* | AAGATCCCACACACGGCTGT | AAACTTGGTCCTCTGATCTCCTCC |  | 141 |
| *SEMA3D* | AACCAGCTCCATCTTCAAAGGCTC | CTTGGACATGTACCAGGCCGT |  | 159 |
| *SEMA3E* | TTTCTTCAAAGCGGCAACAGCT | CTGTTGGGTAATACCGGGAGCA |  | 165 |
| *SEMA3F* | CGATGACGGTGGTCACTGTTG | CAGCGTAAATGACAGGGTTCCT | 58 | 169 |
| *SEMA3G* | TGACCAGCTAGAGGATGTGTTCC | CAAAGGGCCCGTTGAAAACCT | 62 | 155 |
| *NRP1* | ATCACGTGCAGCTCAAGTGG | TCATGCAGTGGGCAGAGTTC | 60 | 168 |
| *NRP2* | GGATGGCATTCCACATGTTGGC | ACCAGGTAGTAACGCGCAGAG |  | 153 |
| *PLXND1* | GTACCCAGGCATGATCCTGC | TTGCAGTAGGCAATCTGGTGAC |  | 141 |
| *PLXNA2* | ACCAACTGTGCCTGTCCTGT | GCTGGGGACAGTCCTCTGAAAT |  | 135 |
| *ITGΒ1* | GCGTGCAGGTGCAATGAAGG | ACAAACACACTGTCCGCAGACG |  | 153 |
| *ITGΒ3* | TTGGAGACACGGTGAGCTTCAG | CTGGCAGGCACAGTCACAATC |  | 143 |
| *ITGΑ5* | GTGGCCTGCATCAACCTTAGC | TTCTGGATGAGCAGGGTCTGG |  | 176 |
| *ITGΑV* | GCGTATCTGCGGGATGAATCTG | AATGTTAGCAGGCGTGAACTGG |  | 141 |
| *CDH1* | ACAACGACCCAACCCAAGAATC | GTCACACACGCTGACCTCTAAG |  | 137 |
| *CDH2* | GTCACCGTGGTCAAACCAATCG | CACGGTTGCAGTTGACTGAGG |  | 123 |
| *VEGFA* | AGGAGTCCAACATCACCATGCA | CAAGGCCCACAGGGATTTTCTTG |  | 145 |
| *KDR* | CATCGCGAAAGTGTATCCACAGG | TTCAAAGGGAGGCGAGCATC |  | 151 |
| *ACTB^*^* | AGAGCTACGAGCTGCCTGAC | AGCACTGTGTTGGCGTACAG |  | 184 |
| *GAPDH^*^* | TCAAGATCATCAGCAATGCCT | CATGAGTCCTTCCACGATACC |  | 95 |

*Housekeeping genes

Some of gene primer sequences were chosen based on published data: *SEMA3A* [Kamata et al., 2015], *SEMA3F* [Gao et al., 2015], and *NRP1* [Lantuéjoul et al., 2003]. Other primer sequences were created in our laboratory.
